# Sustained effects of single doses of classical psychedelics in humans

**DOI:** 10.1038/s41386-022-01361-x

**Published:** 2022-06-21

**Authors:** Gitte M. Knudsen

**Affiliations:** Neurobiology Research Unit, Rigshospitalet and Department of Clinical Medicine, University of Copenhagen, Copenhagen, Denmark

**Keywords:** Outcomes research, Perception

## Abstract

The serotonergic classical psychedelics include compounds that primarily activate the brain’s serotonin 2 A receptor (5-HT2AR), such as LSD, psilocybin, and DMT (ayahuasca). The acute effects of these compounds are well-known as are their ability to increase the emotional state both in healthy people and in those with neuropsychiatric disorders. In particular psilocybin, the psychoactive constituent in “magic mushrooms”, has shown great potential for treatment of anxiety and depression. A unique and compelling feature of psychedelics is that intake of just a single psychedelic dose is associated with long-lasting effects. This includes effects on personality, e.g., higher openness, and amelioration of depressive symptoms. This review focuses on these stunning effects and summarizes our current knowledge on which behavioral, biochemical, neuroimaging, and electrophysiological data support that the intriguing effects of psychedelics on the human brain and mind are based on neural plasticity. The review also points to so far understudied areas and suggests research questions to be addressed in future studies which potentially can help to understand the intriguing long-term effects after intake of a single (or a few) psychedelic doses.

## Introduction

A unique and compelling feature of classical psychedelics is that intake of just a single psychedelic dose is associated with long-lasting (i.e., weeks-years) effects in humans. These include effects on behaviors, attitudes, values, and personality, i.e., elements of a human’s individuality that normally are regarded as relatively stable throughout adulthood. Moreover, these effects are apparent not only in healthy individuals but also in patients diagnosed with various neuropsychiatric disorders, most notably depression and anxiety, who may experience amelioration of their depressive and anxious symptoms. The evidence for the effects is reviewed below; here, acute effects are defined as those present while the drug is still present in plasma and long-term effects are those observed after 1 week, or later.

In a ten-year follow-up study including 247 individuals, intake of LSD, whether with or without a psychotherapeutic setting, resulted in positive personality changes but only in the 23% who subsequently used LSD again [1]. A smaller but controlled study of 16 healthy individuals who were followed-up after 12-months found no changes in personality [2]. In another better controlled longitudinal study of 52 psychedelic-naïve healthy participants who underwent up to four session involving varying doses of psilocybin, the personality trait Openness (as measured with NEO-PI) was increased 1–2 months and 14 months after the intervention [3]. In a subset of this cohort, individuals also reported on externally validated positive changes in attitudes, mood, and behavior 14 months later, with the ascending dose sequence showing greater positive effects [4] and this has since been replicated in several studies [5], including a large web-based study involving different psychedelics [6]. The observation of long-lasting effects on Openness after a single dose of psilocybin in healthy individuals has subsequently been replicated in, e.g., [7]. Compared with placebo, psilocybin also enhances mindfulness and improves psychosocial functioning at 3-4-month follow-up [8].

Data on personality and well-being from classical psychedelics other than psilocybin are more limited. In a placebo-controlled study in 20 healthy volunteers, Openness was significantly increased 2 weeks post LSD [9] whereas a similar trial in 16 healthy individuals did not find significant changes in Openness one and 12 months after LSD [2]. On the other hand, the latter study did reveal higher positive attitudes about life and/or self, positive mood changes, social effects, and behavioral changes, and well-being/life satisfaction both at 1 and 12 months [2]. Ayahuasca (consisting of N,N-Dimethyltryptamine (DMT) and a monoamine oxidase inhibitor) also seems to produce less consistent effects on personality; Openness 3 weeks post-drug intake increased in only one of two trials [10], but relative to baseline, it enhanced emotional and cognitive processes, lasting up to 4 weeks after the experience [11, 12]. It should be mentioned that some of these studies were observational and far from all placebo controlled. The field of psychedelic research is particularly prone to suffer from inability to appropriate blinding and by study participants often being biased towards use of psychedelics. The psychological effects of psychedelics in healthy controls and patient groups were recently assessed in a systematic review and meta-analysis [13] which also identified the need for careful, large-scale, placebo-controlled randomized trials.

Given that moderate-high doses of psilocybin are required to induce lasting changes in personality and mood, does the *content* of the experience then matter? Such observations would be important to understand whether the psychedelic experience is a prerequisite for long-term effects. In a seminal paper, it was noted that participants who had so-called mystical experiences during their psilocybin session, Openness remained significantly higher than baseline more than one year after the session and the change was correlated to the intensity of the mystical experience [3]. There is also additional evidence that the strength of mystical experience may correlate with therapeutic effects in smokers [14] and with diminished anxiety and depression in terminal cancer patients [15–17]. Common dimensions in mystical experiences include the experience of profound unity with all that exists, a felt sense of sacredness, a sense of the experience of truth and reality at a fundamental level, deeply felt positive mood, transcendence of time and space, and difficulty explaining the experience in words, this has been assessed with different versions of the Mystical Experience Questionnaire (MEQ), developed and validated based on psilocybin sessions [18]. Interestingly, we have observed in our studies that those individuals who have a psilocybin-elicited mystical experience (roughly half of them) also tend to have a mystical experience when sessions are repeated later (unpublished observation). These as well as other observations have fostered a functional neural model of mystical experience [19].

A separate question is if these psychedelics-associated effects on personality and mood in healthy individuals are mechanistically related to the therapeutic effects reported in patients with depression or anxiety. There is some data in support of that view: In 20 patients with moderate or severe, unipolar, treatment-resistant depression (TRD), psilocybin (10 and 25 mg, one week apart) lead to an increase in Openness and an increase in Extraversion at 3-month follow-up [20]; this observation should be seen in the light of observed increase in Neuroticism and decrease in Extraversion found in patients with seasonal affective disorder when comparing their depressed state to their symptom-free states [21]. The psilocybin-associated increase in Openness might thus constitute an effect more specific to psychedelic therapy. Cognitive flexibility, broadly defined as the ability to adaptively switch between different cognitive operations in response to changing demands, is a core characteristic of Openness [22] and is often impaired in major depressive disorder (MDD). It has indeed been found that patients with MDD who are treated with psilocybin have increased cognitive flexibility for at least 4 weeks after [23].

Psychedelic therapy for psychiatric disorders is also unique in that the effects are instantaneous after the first session and to the extent that there are follow-up data beyond 6–12 months, it does not (always) require additional sessions to maintain the effect beyond that observation period. In a recent review of 10 independent psychedelic-assisted therapy trials (7 with psilocybin, 2 with ayahuasca, and one with LSD), including patients with anxiety, depression, obsessive-compulsive or substance abuse disorders, the therapeutic effects appeared to be long-lasting (3 weeks - 6 months) after only 1 to 3 treatment session(s) [24]. New studies are continuously added, e.g., a recent study where ayahuasca was given to patients with depression shows beneficial effects, lasting for more than a year [25].

How do these personality and mood effects of psychedelics in humans then back-translate to animals? That could be the topic of whole other review, but it suffices here to say that the drug-induced head twitch response (HTR), commonly taken as a proxy for acute psychedelic effects in animal, does require comparably larger psychedelic doses in animals and generally, most animal studies do involve larger drug doses. Since psychedelic effects of psilocybin in humans correlate with 5-HT2AR occupancy and plasma psilocin levels [26] we have previously suggested that in order to compare animal behavior to human psychedelic effects [27], one should use doses that also generate a 5-HT2AR occupancy of 40–70% in animals.

The neurobiological mechanism behind the stunning psychological long-term action of classical psychedelics has recently become a key question, that mostly has been investigated in animal studies, as reviewed in, e.g., [28]. The findings point to psychedelics inducing molecular and cellular adaptations related to neuroplasticity and these are suggested to occur in parallel to and potentially underly the clinical effects of psychedelics. A relevant question is if epigenetic-driven changes in synaptic plasticity are the mechanistic substrate of psychedelic’s long-lasting actions in humans; data on this topic is also starting to emerge [29].

The remaining part of this review will address how the effects of psychedelics on the neuroplasticity measured observed in animal studies can be tested in humans. Although the methodologies that can be applied to study molecular, structural or functional neuroplasticity in humans are more limited, the evidence for neuroplastic effects taking place and potentially explaining some of the beneficial effects in humans are summarized below.

## Blood measures of neuroplasticity

Molecular changes as part of neuronal plasticity (signaling pathways, gene expression, and protein synthesis) are difficult to assess in vivo in the human brain but Brain Derived Neurotrophic Factor (BDNF) or other neurotrophic factors can be measured in serum or whole blood [30]. Plasma and cerebrospinal fluid BDNF in humans are, on the other hand, considered too low to be reliably determined and an association between blood and brain BDNF remains to be shown in humans. In support of using serum or whole blood BDNF as a proxy for brain BDNF levels, however, is the evidence that across species, peripheral measures of BDNF reflects the brain tissue content [31].

Serum or blood BDNF is lower in patients with MDD [32] and most studies show that treatment for several weeks with selective serotonin reuptake inhibitors (SSRIs) increases serum BDNF [33–35], in line with the hypothesis of SSRI’s also acting through neuroplastic changes. The temporal BDNF response to classical psychedelics is less well studied. Two studies investigated the acute effects of LSD on BDNF: When low doses of LSD (5, 10, and 20 μg) were given to healthy volunteers, plasma BDNF increased at 4 h (5 μg) and 6 h (5 and 20 μg), compared to placebo [36]. When higher doses of LSD (25, 50, 100, and 200 µg) were given, plasma BDNF levels were increased up to 12 h after and only at the 200 µg LSD dose [37]. Both these studies did, however, analyze BDNF in plasma samples which as mentioned above is considered suboptimal since the low levels makes it difficult to measure it accurately and even a minor leakage from the thrombocytes can confound the BDNF measurement. A single dose of ayahuasca also increased serum BDNF levels 48 h after in the active compared to the placebo group; in both healthy controls and patients with TRD [38].

In summary, although there is some data to support that peripheral BDNF is increased up to 12 h after LSD and 48 h after ayahuasca, its eventual relevance for emergence of long-term effects on personality and mood is unclear. It is also unknown how long after the psychedelic drug intervention BDNF remains elevated.

## Molecular, structural and functional neuroplasticity: Neuroimaging studies

### Positron emission tomography (PET)

The classical psychedelics LSD, psilocin and DMT (the psychedelic component of ayahuasca) also target other receptors than 5-HT2AR [39]. There is little doubt, however, that stimulation of the neocortical 5-HT2AR is a requirement for the psychedelic experience to occur. Blocking 5-HT2A/5-HT2C receptors with the 5-HT2AR antagonist ketanserin abolishes virtually all of the subjective effects of subsequently given psilocybin, LSD, and DMT in humans [40] and there is a tight correlation between plasma psilocin, cerebral 5-HT2AR occupancy as measured with PET, and the perceived intensity of the psychedelic experience [26]. This means that measuring individuals’ plasma psilocin levels can give a good estimate of the brain 5-HT2AR occupancy which facilitates comparisons across individuals [41].

As mentioned above, increased Openness is one of the key features of long-term effects. Interestingly, neocortex 5-HT2AR binding as measured with PET has been found to be negatively associated with both the peak plateau duration of the psychedelic experience and with the mystical experience total score [42] meaning that individual differences in baseline cerebral 5-HT2AR may make individuals more prone to the positive/therapeutic effects of psychedelics. In 10 psychedelic-preferring recreational users, 14 MDMA-preferring users and 21 non-using controls, Openness scores differed between the three groups; psychedelic-preferring recreational users showing higher Openness compared with both MDMA-preferring users and controls [43]. Openness scores were positively associated with lifetime number of psychedelic exposures, and among all MDMA-preferring user/psychedelic-preferring recreational user individuals, frontal serotonin transporter - but not frontal 5-HT2AR binding - was positively associated with Openness [43]. Regular use of psychedelics could also matter: Regular users of classical psychedelics have lower neocortex 5-HT2AR than non-users [44] but since these observations are cross-sectional, it is difficult to determine if cerebral 5-HT2AR is a trait or state marker. In other words, are the psychedelic recreational users more prone to use psychedelics because of their lower 5-HT2AR, or does the 5-HT2AR downregulate in response to use of psychedelics? There is some data to support the latter: A single psilocybin dose leads to increased mindfulness as measured 3 months later, preceded by a proportional relative decrease in neocortical 5-HT2A receptor binding [7].

More recently, it has become possible to conduct PET imaging of the synaptic vesicular protein 2A (SV2A) in vivo in humans and there is some evidence to support that SV2A is an alternative synaptic density marker to synaptophysin [45]. In a small study of a heterogeneous group of patients with depressive symptoms, lower presynaptic binding in terms of SV2A was found in prefrontal cortex and hippocampus [46] and SV2A has also been found to be upregulated in the pig brain 1 and 7 days after the pig was given a single psychedelic dose of psilocybin [47]. There are so far no data available from studies in humans who have taken psychedelic drugs.

### Magnetic resonance spectroscopy (MRS)

It is generally believed that extensive release of γ-aminobutyric acid or glutamate causes dendritic spine formation [48]. 5-HT2AR agonists such as the classical psychedelics are excitatory and the agonist action of these substances on 5-HT2A receptors expressed in frontal and limbic areas increases glutamatergic transmission and thereby potentially neuroplasticity, as reviewed in [49]. In consistency with this, a MR spectroscopy study conducted in healthy humans under influence of psilocybin shows that glutamate is released, at least in certain brain regions [50] whereas in patients with MDD, glutamate is reduced in the anterior cingulate but not in hippocampus, when comparing pre- versus one week after psilocybin intake [23]. It would be interesting to know if the extent to which glutamate is released and subsequently decreased is correlated to long-lasting psychological effects, but there are not yet data to answer that question.

### Structural MRI

Measures of structural plasticity in terms of hippocampal volume can be assessed with structural brain MRI. As shown in a meta-analysis of 32 MRI studies [51], MDD patients has in many, but not all, studies been shown to have hippocampal atrophy. Moreover, non-pharmacological treatment of depression with electroconvulsive therapy leads to an increase in hippocampal volume weeks after as compared to pre-treatment volume; this is documented in a literature review and meta-analysis of 17 studies [52, 53]. So far, no published studies have described the long-term effects of psychedelic therapy on hippocampal volume in patients with MDD or in healthy controls.

### Task-based functional magnetic resonance imaging (fMRI)

Whereas a number of task-based fMRI studies have investigated the acute effects of psychedelics [54], only a few of them have done follow-up studies or related the fMRI outcome to long-term effects. In a longitudinal study with psilocybin, healthy volunteers were assessed in an open-label pilot study with fMRI examinations the day before, one week after, and one month after receiving a 25 mg/70 kg dose of psilocybin [5]. One-week post-psilocybin, negative affect and amygdala response to facial affect stimuli were reduced, whereas positive affect and dorsal lateral prefrontal and medial orbitofrontal cortex responses to emotionally-conflicting stimuli were increased. One-month post-psilocybin, negative affective and amygdala response to facial affect stimuli had returned to baseline levels while positive affect remained elevated, and trait anxiety was reduced. In future studies, it will be interesting to see if such findings not only can be replicated but also if they represent more stable biomarkers for neuroplastic effects.

### Resting-state functional connectivity (RSFC)

Functional MR RSFC measures correlations between blood-oxygen-level-dependent (BOLD) signals in individuals instructed to let their mind wander [55]. Over the last years, a large range of novel neurocomputational models have been developed and employed to analyze datasets from patients and healthy controls undergoing psychedelic sessions. This complicates between-studies comparisons and, in particular, replications. Moreover, as shown in a recent review [56], 24 out of the so far 42 published RSFC studies have employed only two out of the 17 unique datasets. Most of these studies involve MR-scanning in the acute or sub-acute psychedelic phase which makes it difficult to determine if eventual changes persist over longer periods, e.g., months. Only a few of the studies included follow-up data.

A single dose of psilocybin can have lasting effects on RSFC in healthy individuals: The number of significant resting-state functional connections across the brain increased from baseline to 1-week and 1-month post-psilocybin [5]. There is also evidence that RSFC is increased 24 h after intake of ayahuasca [57]. Another study shows that one week and 3 months after a psilocybin session, the executive control network RSFC remains decreased compared to pre-intervention [58] and changes in brain network integrity and segregation correlate with both plasma psilocin level and psychedelic experience. Interestingly, the degree to which the change in neocortex 5-HT2AR and RSFC was decreased one week after predicted increased mindfulness 3 months later [59]. Consistent with this observation, psilocybin decreased ECN RSFC in unmedicated, first-time MDD patients with hyper-connectivity between the left dorsolateral prefrontal cortex and frontal and parietal regions, nodes which commonly constitute cognitive control networks [60]. However, a meta-analysis based on 27 seed-based voxel-wise RSFC data sets concluded that MDD is characterized by reduced frontoparietal control system connectivity [61]. In the future, it would be valuable to investigate RSFC longitudinally with a consensus-based methodological battery of tools, involving larger cohorts of both MDD patients and controls and with appropriate measures of replication. As for task-based fMRI, it will be interesting to see if RSFC represents a more stable biomarker for neuroplastic effects.

## Visual evoked potentials for measuring Long-term potentiation (LTP)

Long-term potentiation (LTP), a form of Hebbian neuroplasticity, is characterized by enhanced synaptic efficacy, and it is considered the prime candidate to be the cellular correlate to experience-dependent learning [62]. This type of synaptic plasticity is regulated with BDNF as the primary regulator and it alters the neuron’s structure and its functional properties. The long-term effects of psychedelics on LTP have been electrophysiologically documented in animal studies [28]. In humans, LTP and synaptic plasticity can be assessed with so-called *visual LTP* [63] which consists of rapid repetitive presentation of a visual checkerboard (a photic ‘tetanus’) leads to a persistent enhancement of one of the early components of the visual evoked potential, as measured with electroencephalography (EEG). Also other types of sensory stimulation which induce LTP have allowed translation from invasive studies in animals to non-invasive human investigations [64]. In this way, it has been shown that LTP-based neural plasticity increases within the time frame of the antidepressant effects of ketamine in humans, supporting the hypothesis that changes to neural plasticity may be key to the antidepressant properties of ketamine [65]. No studies have so far investigated the effects of classical psychedelics on visual LTP.

## Summary and suggested future research

As should be clear from the present review, there are substantial knowledge gaps to cover before one can confidently establish the mechanistic relationship between exposure to single doses of classical psychedelic drugs and long-term beneficial/therapeutic effects in both healthy humans and in patients with psychiatric disorders. It should also be noted that neuroplasticity per se may not always be beneficial.

Firstly, there is a need for large-scale, placebo-controlled randomized trials of personality, cognition, and other long-term effects with several assessments over at least 12 months. The studies should include a careful evaluation of the qualitative aspects of the psychedelic experience and plasma drug levels should be monitored throughout the session, to ensure interindividual comparability. In such studies, it should be verified if serum peripheral BDNF (and potentially other neurotrophic factors) are increased after psychedelic drug doses. Further, the relationship between the temporal profile of serum BDNF within the first week of exposure and long-term effects should be analyzed. Likewise, the temporal and regional profile of brain glutamate release, changes in brain volume, perturbations in task-related fMRI and in RSFC would benefit from being scrutinized in relation to short- and long-acting classical psychedelics. Common to many of the points raised above is that knowledge about the temporal profile of the different measures can generate a more complete picture of what it takes to induce effects lasting for months, or more. This can generate testable hypotheses about the mechanisms, e.g., neuroplastic changes, behind the effects. Is there for example a time window of a minimum duration where it is critical that certain phenomena take place (e.g., increase in BDNF, mystical experience, RSFC dissolution, cerebral 5-HT2AR reduction, etc.)? And along that line, will short-acting classical psychedelics, e.g., intravenous psilocin work as well as long-acting, e.g., peroral LSD?

Whereas the correlation between plasma drug, cerebral 5-HT2AR occupancy, and the perceived intensity of the psychedelic experience is well-established for psilocybin, these associations should also be established for LSD and DMT. This would ensure that different psychedelic drugs are compared at the same level of 5-HT2AR occupancy, something that would also be of importance for animal studies. Such studies could also help to clarify if different psychedelics have the same effect at comparable occupancy, as agonists may have different efficacy or different functional selectivity [3, 4]. It will also be interesting to see if a proxy for synaptic density, SV2A (as measured with PET), increases in response to classical psychedelics. Another promising technology that lends itself to investigations of neuroplastic effects in humans is visual LTP.

The interindividual variability in response to psychedelic drugs, both in terms of qualitative aspects of the experience (e.g., mystical experience) as well as pre-existing traits, such as Openness, is intriguing and it needs to be established if these traits contribute to drug expectations or through some other mechanism, if at all. The relation between interindividual response and cerebral 5-HT2AR density is also important to clarify.

In conclusion, there are multiple research questions to answer before the exciting observations about neuroplastic changes that take place in animals also are at play for the beneficial and therapeutic effects in humans.
